# Pathological prognostic markers in central nervous system solitary fibrous tumour/hemangiopericytoma: Evidence from a small series

**DOI:** 10.1371/journal.pone.0203570

**Published:** 2018-09-05

**Authors:** Luca Bertero, Vittorio Anfossi, Simona Osella-Abate, Maria Giulia Disanto, Cristina Mantovani, Francesco Zenga, Roberta Rudà, Diego Garbossa, Riccardo Soffietti, Umberto Ricardi, Mauro Papotti, Paola Cassoni

**Affiliations:** 1 Pathology Unit, Department of Medical Sciences, University of Turin, Turin, Italy; 2 Radiation Oncology Unit, Department of Oncology, University of Turin, Turin, Italy; 3 Neurosurgery Unit, Department of Neurosciences, University of Turin, Turin, Italy; 4 Neuro-oncology Unit, Department of Neurosciences, University of Turin, Turin, Italy; 5 Pathology Unit, Department of Oncology, University of Turin, Turin, Italy; George Washington University, UNITED STATES

## Abstract

**Background:**

Primary central nervous system (CNS) solitary fibrous tumour/hemangiopericytoma (SFT/HPC) is a rare neoplasm and its classification criteria have been redefined by the latest WHO Classification of CNS Tumours. Outcome can vary significantly among patients, thus reliable prognostic markers are warranted.

**Methods:**

Primary CNS SFT/HPC diagnosed at the Pathology Unit of our Institution between 2006 and 2016 were retrospectively collected. Tumour grade along with immunohistochemistry for Ki67, STAT6, PHH3, CD34 and Bcl-2 were assessed. TERT promoter status was evaluated by Sanger sequencing.

**Results:**

Fifteen SFT/HPC were analysed: 9/15 (60%) female, median age at diagnosis 60 (range: 10–67). Six (40%) cases showed a SFT phenotype and mean H&E-mitotic count was 4.8/10 HPF. Tumour grade was I in 6, II in 4 and III in 5 cases. Mean PHH3-mitotic count was higher than H&E count (8.4 versus 4.8/10 HPF), but it would have determined a change in tumour grade in a sole case. Nuclear staining for STAT6 was present in 14/15 (93.3%). CD34 and Bcl-2 expression rates were lower in higher grade tumours. TERT promoter was mutated in two cases. Median follow up time was 2.4 years (6 months-7.4 years) and 5/15 (33%) patients developed local disease recurrence. Partial resection (p = 0.0185), higher WHO grade (p = 0.038), lower CD34 (p = 0.038) and Bcl-2 (p = 0.010) expressions were significantly associated with a poorer disease-free interval.

**Conclusions:**

WHO grade is the main prognostic tool in CNS SFT/HPC, but it could be integrated by other markers, like CD34 and Bcl-2, in the clinical practice. The relevance of TERT promoter mutations in this subset of CNS tumours needs further evaluation.

## Introduction

Classification of primary CNS solitary fibrous tumour/hemangiopericytoma (SFT/HPC) has been recently redefined by the latest World Health Organization (WHO) Classification of Tumours of the Central Nervous System (CNS)[1] by merging into a single category two previously separated diagnostic entities (solitary fibrous tumour and hemangiopericytoma). This change mirrors what was already accepted for SFT/HPC of other sites (e.g. soft tissues and pleura) and is supported by a shared molecular hallmark: the chromosomal inversion at the 12q13 locus, fusing the NGFI-A-binding protein 2 (*NAB2*) and the signal transducer and activator of transcription 6 (STAT6) genes *[2, 3]*. This alteration is indeed present in most SFT/HPC cases regardless of tumour site or grade. NAB2-STAT6 fusion protein promotes tumour growth by localizing to the nucleus and activating the *EGR* gene. Nuclear localization of the fusion protein can be detected by anti-STAT6 antibodies[4] and STAT6 immunohistochemistry (IHC) is currently a recommended diagnostic tool by the latest WHO classification, since no nuclear STAT6 staining is present in meningiomas or in other frequent meningeal tumours, thus helping in the differential diagnosis.[1, 4]

CNS SFT/HPC are rare (less than 1% of all primary CNS tumours), usually dural-based intracranial tumours, thought to develop from CD34-positive fibroblasts present in the dura mater or in the intraparenchymal perivascular connective tissue[5, 6] and mostly occur in adults, with a slightly higher incidence in males.[7] Exact epidemiological data are difficult to ascertain, due to both their rarity and to the changes of classification criteria over time.

Prognosis is often good, especially in cases with the solitary fibrous tumour morphology and when gross total resection is achieved; conversely, the hemangiopericytoma morphology is associated with a higher rate of local recurrences (>75% in patients with long term follow up) and distant metastases occurrence.[8–12] Based on these findings, the current WHO grading criteria provide that SFT/HPC with solitary fibrous tumour phenotype and mitotic count <5 mitoses/10 high-power fields (HPF) are classified as grade I, whereas tumours with hemangiopericytoma morphology are graded as grade II or III based on the mitotic count with a cut-off value of 5 mitoses/10 HPF.[1] Radiotherapy may be of benefit to patients with incomplete resection or at higher risk of recurrence.[13, 14]

Overall, compared to extra-CNS SFT/HPC, prognostic markers are less defined and available data are limited by the rarity of this tumour; thus, new and reliable prognostic factors are warranted to help personalize patients’ therapeutic management. Therefore, we wanted to retrospectively assess novel possible prognostic factors in a series of SFT/HPC diagnosed at our Institution, evaluating both histopathological and molecular features. In particular, we evaluated the prognostic relevance of two IHC markers: CD34 and B-cell lymphoma 2 (Bcl-2), which, although non-specific, can be useful diagnostic tools and were especially important in the pre-STAT6 era.[15] CD34 loss has been reported as a characteristic finding in dedifferentiated extra-CNS SFT.[16] Secondly, considered the prognostic relevance of mitotic count in this tumour entity, we wanted to evaluate if a count based on phosphorylated histone H3 (PHH3) IHC could improve its grading and prognostic capability compared to H&E-based mitotic count. PHH3 antibody specifically recognizes the phosphorylated (at serines 10 and 28) histone H3, which is present in cells at the end of G2 or in the M phase of cell cycle and it has already been validated as a useful IHC marker to improve mitotic count sensitivity in different types of neoplasms, including breast cancer and meningiomas.[17, 18] In terms of molecular profile, telomerase maintenance has been recently described as a hallmark of most human cancers, needed by neoplastic cells to escape replicative senescence. *TERT* gene codes for the catalytic subunit of the telomerase complex and recurrent point mutations of its promoter have been shown to create a novel binding site for E-twenty-six (ETS) family transcription factors, leading to increased telomerase activity in affected cells. *TERT* promoter mutations are common to different malignancies, with variable prognostic significance.[19–22] Since this molecular alteration seems to be associated with adverse outcome in extra-CNS SFT,[23] we wanted to explore its possible prognostic meaning in our series.

## Materials and methods

### Case series and tissue samples

Fifteen consecutive cases of primary CNS solitary fibrous tumours or hemangiopericytomas diagnosed at the Pathology Unit of City of Health and Science University Hospital of Turin between 2006 and 2016 were retrospectively collected. All patients underwent surgical resection at the Neurosurgery Unit of the same Institution. Follow up data were retrieved from patients’ charts. The study was conducted in accordance with The Code of Ethics of the World Medical Association (Declaration of Helsinki) for experiments involving humans and within guidelines and regulations by the Research Ethics Committee of the University of Turin. Written informed consent was not obtained because patients were no longer undergoing regular follow up visits directly at our institution or were deceased; considered the retrospective nature of the present study and that it had no impact at all on patients’ care, verbal informed consent was obtained from participants or their relatives and recorded in a specific log. This study, including the verbal consent procedure, was approved by the Research Ethics Committee of the University of Turin.

### Morphology and immunohistochemistry

All diagnoses were confirmed by a senior neuropathologist (PC) and reassessed according to the 2016 WHO classification. Thus, tumours were graded based on their phenotype (solitary fibrous tumour versus hemangiopericytoma) and mitotic count on H&E slides. Mitotic count was repeated after PHH3 IHC (polyclonal, Ventana Medical Systems Inc., Tucson, AZ, US). IHC for STAT6 (polyclonal, Spring Bioscience Corporation, Pleasanton, CA, US), Ki67 (clone 30–9, Ventana Medical Systems Inc., Tucson, AZ, US), CD34 (clone QBEnd/10, Ventana Medical Systems Inc., Tucson, AZ, US) and Bcl-2 (clone SP66, Ventana Medical Systems Inc., Tucson, AZ, US) were also performed. Immunohistochemistry for Ki67, CD34 and Bcl-2 was performed on all tissue blocks of each case; Ki67 labelling index was evaluated by counting at least 1000 tumour cells while CD34 and Bcl-2 were assessed by visual estimation. Nuclear staining for STAT6 was considered consistent with the SFT/HPC diagnosis. BenchMark ULTRA platform (Ventana Medical Systems Inc., Tucson, AZ, USA) was used for all IHC.

### *TERT* promoter sequencing

DNA extraction from formalin-fixed and paraffin-embedded (FPPE) tumour samples was performed as previously described[24] and concentrations/purity were measured by a Nanodrop 1000 spectrophotometer (Thermo Fisher Scientific, Waltham, MA, USA).

Mutational status of the telomerase reverse transcriptase (TERT) promoter region from position -27 to -286 from ATG start site, including the polymorphic site represented by rs2853669, were determined by PCR and Sanger sequencing using the following primer pair: promoter forward 5′-CAGCGCTGCCTGAAACTC-3′ and reverse 5′-GTCCTGCCCCTTCACCTT-3′, as described by Horn et al.[25] PCR products were purified and used as template for the sequencing reactions, which were performed with BigDye Terminator v1.1 Cycle Sequencing Kit (Applied Biosystems, Foster City, CA, USA). After purification, the sequences were analyzed by Sanger direct sequencing using the ABIPRISM 3130 Genetic Analyzer (Applied Biosystems, Foster City, CA, USA).

### Statistical analyses

Statistical analyses were performed using Stata/MP 15.0 Statistical Software (StataCorp, College Station, TX, USA). Categorical variables were compared using Pearson’s χ^2^ test. Kruskall-Wallis has been used to compare not normally distributed variables. The variables tested were: age at diagnosis, gender, tumour phenotype, grading based on H&E and on PHH3 mitotic count (<5/10 HPF *versus* ≥5/10 HPF), tumour size based on MRI report before surgery (<3 cm *versus* ≥3), Ki67 labelling index (≤5% *versus* >5%), CD34 expression (<80% versus ≥80%), Bcl-2 expression (<30% versus ≥30%), TERT promoter status and tumour recurrence. Differences were considered significant when p < 0.05 for reported two-sided p-values. Survival curves between different groups were plotted using the Kaplan-Meier method and the statistical comparisons were performed with Log-rank test.

## Results

### Clinical characteristics

**Fig 1:**
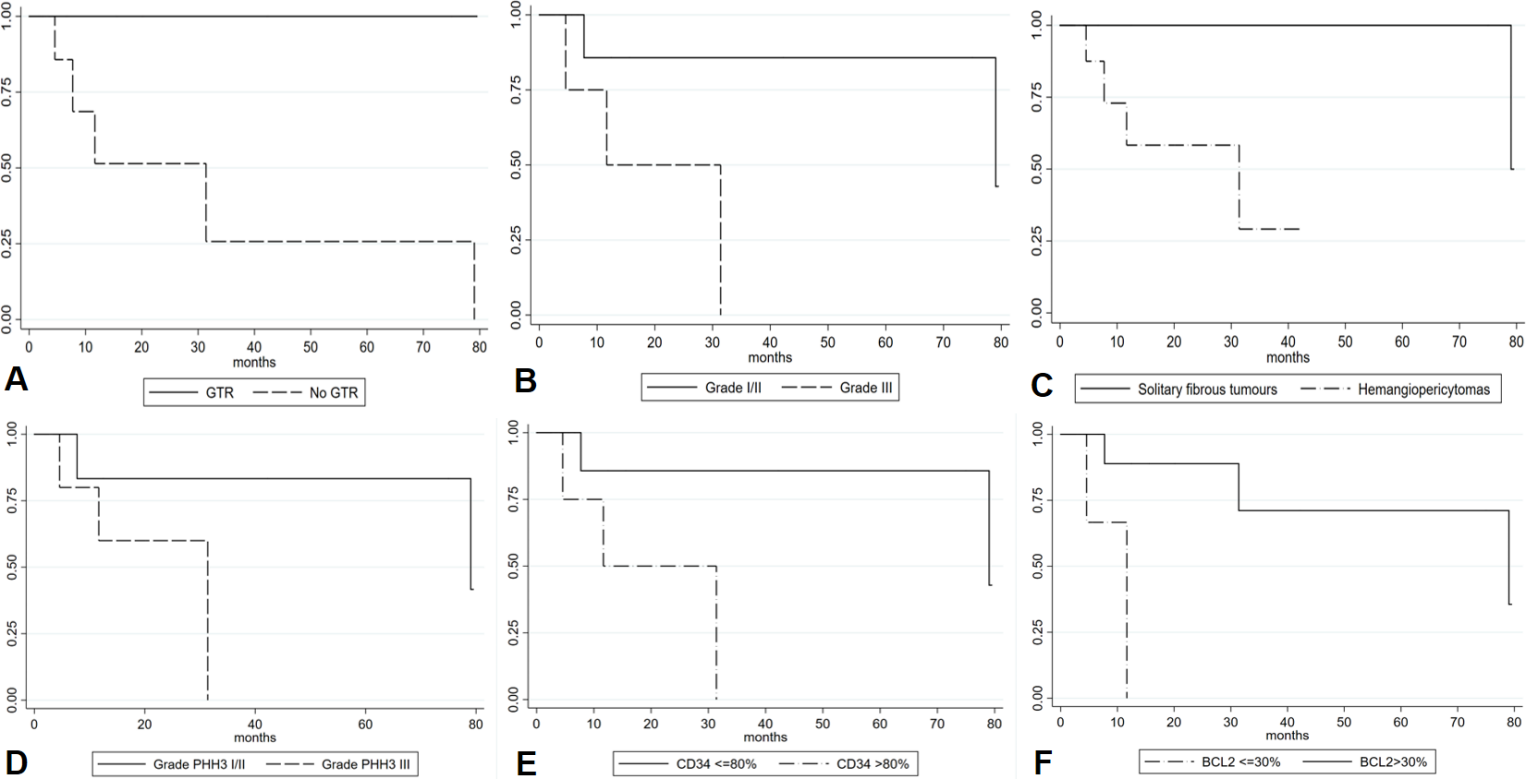
Kaplan-Meier analysis of disease free interval according to different clinico-pathological variables. Gross total resection (log-rank test p = 0.0185) (A), WHO grade (log-rank test p = 0.038) (B), tumour phenotype (solitary fibrous tumour *versus* hemangiopericytoma) (log-rank test p = 0.062) (C), tumour grade according to PHH3-based mitotic count (log-rank test p = 0.094) (D), CD34 expression (log-rank test p = 0.038) (E) and Bcl-2 expression (log-rank test p = 0.010) (F).

Fifteen SFT/HPC cases were retrospectively collected: 9/15 (60%) were female, median age at diagnosis was 60 (range: 10–67). Median follow up time was 2.4 years (6 months-7.4 years). Gender, age and tumour site were not significantly correlated with tumour morphology/phenotype (Table 1). Tumour site was as follows: cranial base 8/15 (53%), cerebral hemispheres 4/15 (27%) and posterior fossa 3/15 (20%). Gross total resection was not achieved in 7/15 (47%) and in 4 of these cases (57%), tumour was located at cranial base. Five patients (33%) developed local disease recurrence, but none showed distant metastases; all patients with disease recurrence had an incomplete surgical resection. Ten patients out of 15% (66%) were alive at end of follow up, whereas 5 patients died (3/5 because of SFT/HPC progression, 2/5 due to other causes). Gross total resection was significantly associated with a more favourable disease-free interval (DFI) (log-rank test p = 0.0185) (Fig 1A), while no correlation was found for age, gender or tumour site.

**Table 1:** Clinico-pathological characteristics according to tumour phenotype.

			Tumour phenotype		p
		Total (n)	Solitary fibrous tumour (n)	Hemangiopericytoma (n)	
**Gender**	**F**	9	4	5	0.667
	**M**	6	2	4	
**Age**	**≤60 years**	8	3	5	0.833
	**>60 years**	7	3	4	
**Tumour site**	**Cerebral hemispheres**	4	1	3	0.526
	**Cranial base**	8	3	5	
	**Posterior fossa**	3	2	1	
**Tumour size**	**<3 cm**	9	4	5	0.667
	**≥3 cm**	6	2	4	
**WHO grade (H&E-based mitotic count)**	**I**	6	6	0	0.001
	**II**	4	0	4	
	**III**	5	0	5	
**WHO grade (PHH3-based mitotic count)**	**I**	6	6	0	0.001
	**II**	3	0	3	
	**III**	6	0	6	
**H&E-based mitotic count**	**<5**	10	6	4	0.044
	**≥5**	5	0	5	
**PHH3-based mitotic count**	**<5** **≥5**	9	6	3	0.028
	**≥5**	6	0	6	
**KI67 labelling index**	**≤5%**	8	6	2	0.003
	**>5%**	7	0	7	
**CD34%**	**<80%**	5	0	5	0.025
	**≥80%**	10	6	4	
**Bcl2%**	**≤30%**	5	1	4	0.264
	**>30%**	10	5	5	
***TERT* promoter mutations**	**Wildtype**	10	5	5	1.000
	**Mutated**	2	1	1	
***TERT* SNP rs2853669**	**No**	6	2	4	0.248
	**Yes**	6	4	2	
**Tumour recurrence**	**No**	10	5	5	0.580
	**Yes**	5	1	4	

### Histological features and grading

**Fig 2:**
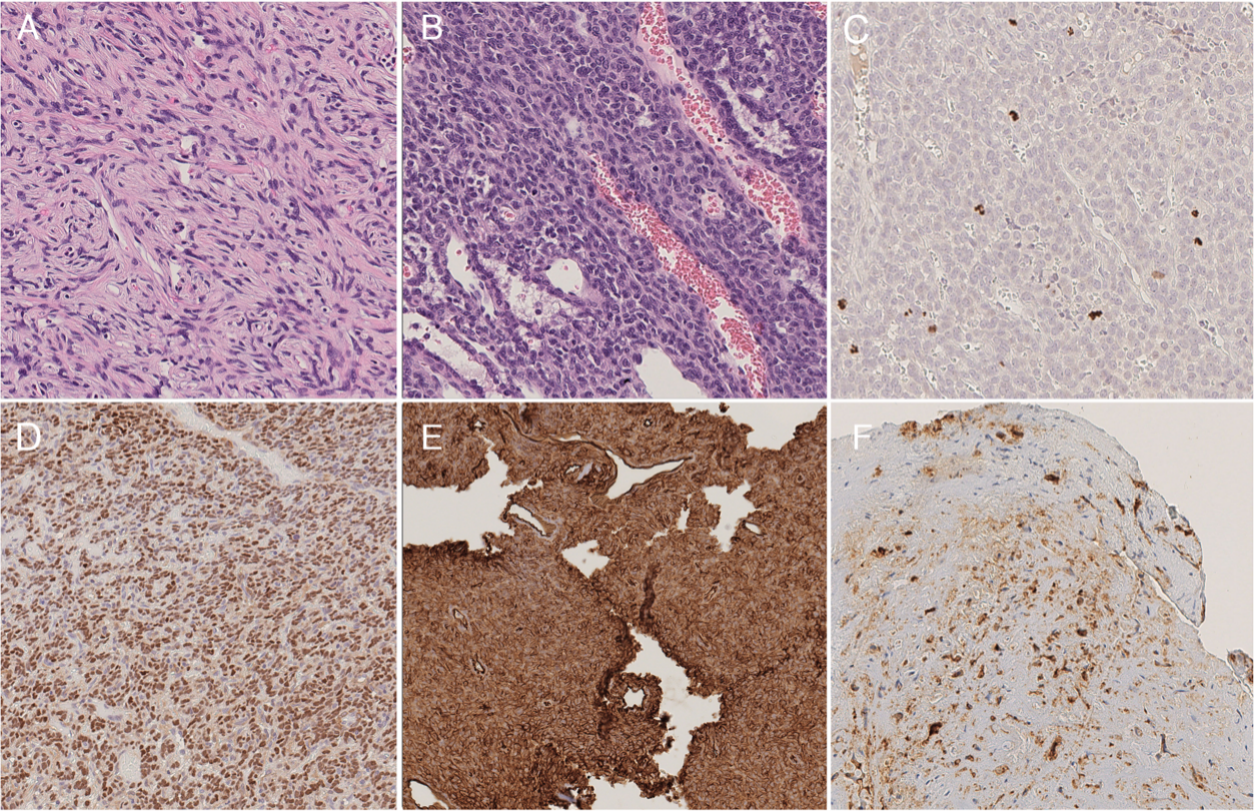
Example images showing the different morphological and immunohistochemical features of the case series. Images of the solitary fibrous tumour (A) and the hemangiopericytoma (B) phenotypes. Mitotic figures highlighted by PHH3 immunohistochemistry (C). STAT6 nuclear staining (D), a characteristic finding of SFT/HPC. Diffuse (E) and focal (F) CD34 positivity.

Six cases showed a solitary fibrous tumour phenotype (6/15, 40%) (Fig 2A, while 9/15 (60%) had a hemangiopericytoma morphology (Fig 2B). Mean number of mitoses/10 HPF on H&E was 4.8 [standard deviation (SD): 5.51]. According to WHO criteria, 6 cases were grade I (40%), 4/15 (27%) grade II and the remaining 5 cases (33%) were grade III tumours. No necrosis was identified in any case. H&E-based mitotic count ≥5/10 HPF was significantly correlated with the hemangiopericytoma phenotype (p = 0.044). WHO grade resulted significantly associated with DFI (log-rank test p = 0.038) (Fig 1B), while tumour phenotype did not (log-rank test p = 0.062) (Fig 1C).

## PHH3-based grading

Although the mean number of mitoses/10 HPF observed following PHH3 IHC staining was higher than in H&E (8.4 versus 4.8, SD: 10.69) (Fig 2C), in one case only the PHH3-based mitotic count would have changed the tumour grade (from grade II to III) according to the WHO criteria. As observed for the H&E-based count, PHH3-based mitotic count ≥5/10 HPF significantly correlated with the hemangiopericytoma phenotype (p = 0.028). WHO grade using PHH3-based mitotic count was not significantly associated with DFI (log-rank test p = 0.094) (Fig 1D).

## Immunohistochemistry

Nuclear staining for STAT6 was present in 14/15 (93.3%) (Fig 2D). The only STAT6-negative case displayed the characteristic features of the hemangiopericytoma phenotype and was partially positive for CD34 (40%). Overall, mean Ki67 labelling index was 9% (SD: 7.79%): 3.5% in grade I, 7% in grade II and 16.8% in grade III tumours. Regarding CD34 expression (Fig 2E and 2F), overall mean number of positive cells was 64% (SD: 35.6%): mean rates of 86%, 90% and 19% positive cells were observed in grade I, II and III tumours, respectively (p<0.001). In 3/5 (60%) recurrent cases CD34 expression was <10%. Bcl-2 overall mean expression was 63% (SD: 38.6%), 74%, 76% and 38% in grade I, II and III tumours, respectively (p = 0.2262). Ki67 labelling index (p = 0.003) and CD34 expression (p = 0.025) were significantly correlated with the hemangiopericytoma phenotype (Table 1). CD34 expression ≤80% was associated with poorer DFI (log-rank test p = 0.038) (Fig 1E) as well as Bcl-2 expression ≤30% (log-rank test p = 0.010) (Fig 1F).

## *TERT* promoter sequencing

*TERT* promoter was successfully analysed in twelve cases out of 15 (80%) (one case was excluded because of lacking adequate material for analysis and in the other two cases sequencing failed). *TERT* promoter resulted wildtype in 10/12 (83%) cases, while 2/12 (17%) were mutated: the recurrent 1,295,228 C>T (C228T) mutation and a 1,295,254 C>T (C254T) mutation positioned respectively at 124 and 150 base pairs upstream of the ATG translational start site of TERT were identified; these mutations were found in a grade I and a grade II SFT/HPC, respectively. The first patient had no disease recurrence (follow up: 73 months), while the second patient had no recurrence too, but died of unrelated causes 5 months after diagnosis. Six out of 12 (50%) cases harboured the rs2853669 single nucleotide polymorphism (SNP). Neither of these findings was associated with tumour phenotype (p = 1.000 and p = 0.248) or DFI (log-rank test p = 0.387 and p = 0.739).

## Discussion

The identification of prognostic markers is a crucial issue for appropriate patients’ management, even more when it applies to rare entities or after changes in the classification criteria. Since SFT/HPC of the CNS well resume both these issues, in the present study we analysed and identified putative prognosticators which could be used in the daily practice. Prognostic factors are especially needed in this setting considered that some of the parameters used for risk-stratification of extra-CNS SFT/HPC, like tumour size, are not applicable in the CNS.[26]

Beginning with clinical variables, GTR was significantly associated with a better DFI, a finding consistent with literature data.[8, 12, 27] As previous studies have found, surgical resection alone is sufficient to cure patients in many cases. Although tumour site was not related to DFI in our series, 57% of cases in which GTR was not achieved were located at cranial base, a site which could possibly hamper a complete resection as reported for other CNS tumours (i.e. meningiomas).[28] Among clinical variables neither gender nor age were related to patients’ outcome.

Focusing on histopathological features, the two different tumour phenotypes (SFT and HPC) were not specifically associated with clinical variables (gender, age and tumour site): as a matter of fact, a distinction based on their clinico-radiological characteristics was not deemed possible even when they were considered different entities.[29] The HPC phenotype was associated with a higher mitotic count, but tumour phenotype alone did not reach statistical significance in terms of DFI; on the contrary, a higher WHO grade was associated with a worse outcome. This finding supports the present WHO grading criteria which take into account both tumour phenotype and mitotic count.

In our series, PHH3-based mitotic count was higher than the H&E-based (as expected), but it would have changed the WHO grade (from grade II to grade III) in one case, only; moreover, grading based on this tool was not associated with a different DFI, therefore suggesting its lack of utility in this specific subset of tumours. These results need to be validated in a larger cohort, but for the time being the use of PHH3-based mitotic count in daily practice is not supported.

Among other IHC markers, STAT6 nuclear staining was detected in all cases except for one (14/15, 93.3%), a finding consistent with literature data.[15] Rare STAT6-negative SFT/HPC are possible and in these cases diagnosis should be based on a careful revision of tumour morphology and using other markers like CD34, Bcl-2 and CD99. In terms of prognostic meaning, we found that a lower CD34-expressions was associated with a significantly poorer outcome in our series. Previous research studies found an association between CD34 loss and higher-grade tumours[30, 31] and decreased CD34 expression is also linked to recurrent tumours, a finding confirmed by our data: we observed a very low (<10%) CD34-expression in most of our recurrent cases (3/5, 60%). Bcl-2 expression (when lower than 30%) was associated with prognosis as well. In our series, only one of the 5 recurrent cases showed discordant CD34/Bcl-2 expression with low CD34 and high Bcl-2 expressions. This finding suggests a relationship between these two markers and is worth being validated in a larger series.

*TERT* promoter mutations have been identified in CNS SFT/HPC, but specific data in this setting are limited.[32, 33] In our cases, we observed a lower overall mutation rate compared to previously reported data (16.7% *versus* 25.6%), a difference even greater when considering the HPC phenotype alone (16.7% *versus* 50%).[32] We did not observe an association between *TERT* promoter mutational status and outcome, but this could probably be explained by the limited sample size. Moreover, one of the two identified mutations (C254T) was still unreported in this tumour entity. We also investigated a common *TERT* promoter SNP (rs2853669): its frequency in our series (50%) is in line with its prevalence in the general population and we did not observe an association with outcome.

In conclusion, WHO grade is the main prognostic tool for CNS SFT/HPC, but our data suggest that it could be integrated in the daily practice by other markers, like CD34 and Bcl-2. Also molecular traits, like *TERT* promoter mutations, could provide valuable information, but their significance in this setting needs further evaluation in larger series.
